# Identification of post-transcriptional regulation reveals complexity in peanut pod development by Direct RNA

**DOI:** 10.1007/s42994-025-00224-5

**Published:** 2025-09-08

**Authors:** Wei Wang, Haosong Guo, Jianxin Bian, Fa Cui, Xiaoqin Liu

**Affiliations:** 1 Peking University Institute of Advanced Agricultural Sciences, Shandong Laboratory of Advanced Agriculture Sciences at Weifang, Weifang, 261325 China; 2 College of Agriculture, Ludong University, Yantai, 264025 China

**Keywords:** Peanut pod, Plant development, Alternative polyadenylation, *N*^6^-methyladenosine

## Abstract

Peanut (*Arachis hypogaea*) is widely cultivated worldwide as an important source of edible vegetable oil and protein. Peanut seed pods develop below ground from a gynophore that forms above ground and then penetrates the soil surface to bury the developing pod. Numerous studies have explored transcriptional regulation during peanut pod development. Here, we explored post-transcriptional regulation, including polyadenylation, alternative splicing, and RNA adenosine methylation (m^6^A), in peanut pods across four developmental stages by performing direct RNA sequencing. This produced 70.43 million long reads with average lengths of 890–1,136 nucleotides (nt) from 12 samples across four developmental stages, yielding a total of 14,627 newly identified transcripts. We detected a negative relationship between poly(A) tail lengths and transcript abundance, with the shortest poly(A) tails at the subterranean peg and expanded pod 1 stages, and longest poly(A) tails at the aerial gynophore and expanded pod 2 stages. Moreover, throughout pod development, from the penetration of the gynophore into the soil to pod enlargement, the splicing machinery utilized more proximal than distal alternative polyadenylation sites in the transcripts. The date showed no correlation between m^6^A modification and gene expression in peanut, but found more transcripts with alternative first and last exon types of alternative splicing events. Transcripts that were differentially abundant across developmental stages were primarily enriched in the Gene Ontology terms photosynthesis, response to oxidative stress, response to auxin, plant-type cell wall organization, and lignin catabolism. This study lays a foundation for revealing the roles of epigenetics and post-transcriptional regulation in pod development in peanut.

**Supplementary Information:**

The online version contains supplementary material available at 10.1007/s42994-025-00224-5.

## Introduction

Peanut (*Arachis hypogaea* L.) is a leguminous allotetraploid plant with diploid wild relatives (Chen et al. 2019). Peanuts are grown worldwide as an economically important source of oil and protein (Zhao et al. 2023). For example, in China peanuts contribute significantly to edible oil supply (Lv et al. 2023). Peanut seed pods develop from an aboveground gynophore; following pollination, this structure penetrates the soil, and the remainder of seed pod development occurs underground. Peanut seed pod development affects the yield and quality of peanuts (Cui et al. 2022a). Therefore, examining the development of this important structure is crucial for improving the productivity and quality of peanuts.

Numerous multi-omics studies have examined peanut pod development. For example, next-generation sequencing (NGS) has been used to investigate different developmental stages, as well as the pod transcriptome (Li et al. 2017; Wan et al. 2017). In addition, other omics studies have explored the peanut pod metabolome (Lv et al. 2022), proteome (Zhao et al. 2015), and microRNA landscape (Gao et al. 2017). However, NGS does not directly detect epigenetic modifications on DNA, nor does it detect post-transcriptional RNA modifications such as polyadenylation. To date, researchers have identified over 160 distinct RNA base modifications with known functions (Boccaletto et al. 2022; Helm and Motorin 2017). For example, *N*^6^-methyladenosine (m^6^A) is a common modification in the eukaryotic transcriptome (Yue et al. 2015). *N*^6^-methyladenosine can be dynamically added by methyltransferases, removed by demethylases, and recognized by methylated reading proteins. In recent years, enzymes related to m^6^A modification in plants have been identified. Studies have found that m^6^A modification regulates plant embryogenesis (Zhong et al. 2008), floral transition (Wang et al. 2022), shoot stem cell fate (Shen et al. 2016a), and stress responses (Hou et al. 2022; Tang et al. 2022).

Direct RNA sequencing (DRS), a technique developed by Oxford Nanopore Technologies for native RNA sequencing, enables the detection of RNA base modifications, poly(A) tail lengths (PALs), and RNA splicing forms (Garalde et al. 2018). Since the emergence of DRS, many studies of RNA modifications have been conducted on animals (Reis et al. 2022), yeast (Tresenrider et al. 2021), nematodes (*Caenorhabditis elegans*) (Li et al. 2020), and plants. For example, DRS identified 28,858 full-length transcripts in *C. elegans*, and analysis of these data showed that the PALs of these transcripts vary during development (Roach et al. 2020). In rice (*Oryza sativa*), specific m^6^A modification patterns were detected across tissues; modified transcripts had elevated abundances and shorter poly(A) tails compared with unmodified transcripts (Yu et al. 2023). In poplar (*Populus trichocarpa*), an increase in the m^6^A ratio of transcripts of wood-forming genes following drought treatment results in a decrease in mRNA abundance and protein accumulation, suggesting that m^6^A may inhibit wood formation under drought stress (Gao et al. 2022). In *Arabidopsis* (*Arabidopsis thaliana*), FIONA1 influences m^6^A levels, controlling the mRNA levels of the flowering-time gene *SUPPRESSOR OF OVEREXPRESSION OF CONSTANS1* (*SOC1*) and its upstream regulators and preventing premature flowering (Xu et al. 2022).

The poly(A) tail is a homopolymeric extension of adenosine located at the 3′ ends of most eukaryotic mRNAs, and DRS provides an efficient way of identifying the true sites of polyadenylation. Studies using DRS revealed that the pre-mRNAs of *SOC1*, *REGULATORY PARTICLE NON-ATPASE 10* (*RPN10*), and *FYVE-domain protein required for endosomal sorting 1* (*FYVE1*) tend to use distal poly(A) sites during 3′ untranslated region (UTR) processing in the *Arabidopsis cpsf30-1* mutant, resulting in lower mRNA abundances. CPSF30 regulates alternative polyadenylation (APA), and the *cpsf30-1* mutant shows late flowering and increased sensitivity to abscisic acid (Song et al. 2021). In the *hikeshi-like protein1* (*hlp1*) mutant, the flowering inhibitor gene *FLOWERING LOCUS C* (*FLC*) is upregulated, leading to a late-flowering phenotype. HLP1 binds directly to poly(A) sites in *FLOWERING CONTROL LOCUS A* (*FCA*) transcripts and promotes the use of distal poly(A) sites, thereby generating functional full-length *FCA* transcripts; this leads to repression of *FLC* expression and thus promotes flowering (Zhang et al. 2015). APA may also affect stress responses, as the frequency of transcripts that use distal poly(A) sites increases under drought stress (Gao et al. 2022).

PAL also affects gene expression (Gao et al. 2022), mRNA stability, translation, and the nuclear export of mature mRNA (Eckmann et al. 2011; Fuke and Ohno 2008; Liu et al. 2021). The distribution of PAL shows distinct patterns in pollen and seeds, with significant enrichment of longer PALs. This might be regulated by the pollen-specific expression of poly(A)binding protein (*PABP*) and is related to the stability of mRNA in pollen (Jia et al. 2022). Although DRS can be used to estimate PAL, there are few reports on the poly(A) tails of plant mRNAs.

Alternative splicing allows the processing of mRNA precursors into one or more mature mRNAs by selecting one or more splicing sites. This increases the diversity of proteins and the potential for selectivity by organisms. Therefore, it plays an important role in plant growth and development, as well as in the responses to biotic and abiotic stresses (Kelemen et al. 2013). Alternative splicing events can be identified by DRS. For example, in Moso bamboo (*Phyllostachys edulis*), a greater number of AS events occur during rapid growth, with intron retention (IR) accounting for the majority of these AS events (Li et al. 2023). 

In this study, we employed Nanopore DRS to examine post-transcriptional regulation during peanut pod development. We revealed complex post-transcriptional regulatory dynamics during this process, including effects on splicing, PAL, APA, and m^6^A modifications. Our findings provide insight into the roles of post-transcriptional modifications in the intricate regulation of peanut pod development.

## Results

### DRS at different stages of peanut pod development

**Fig. 1:**
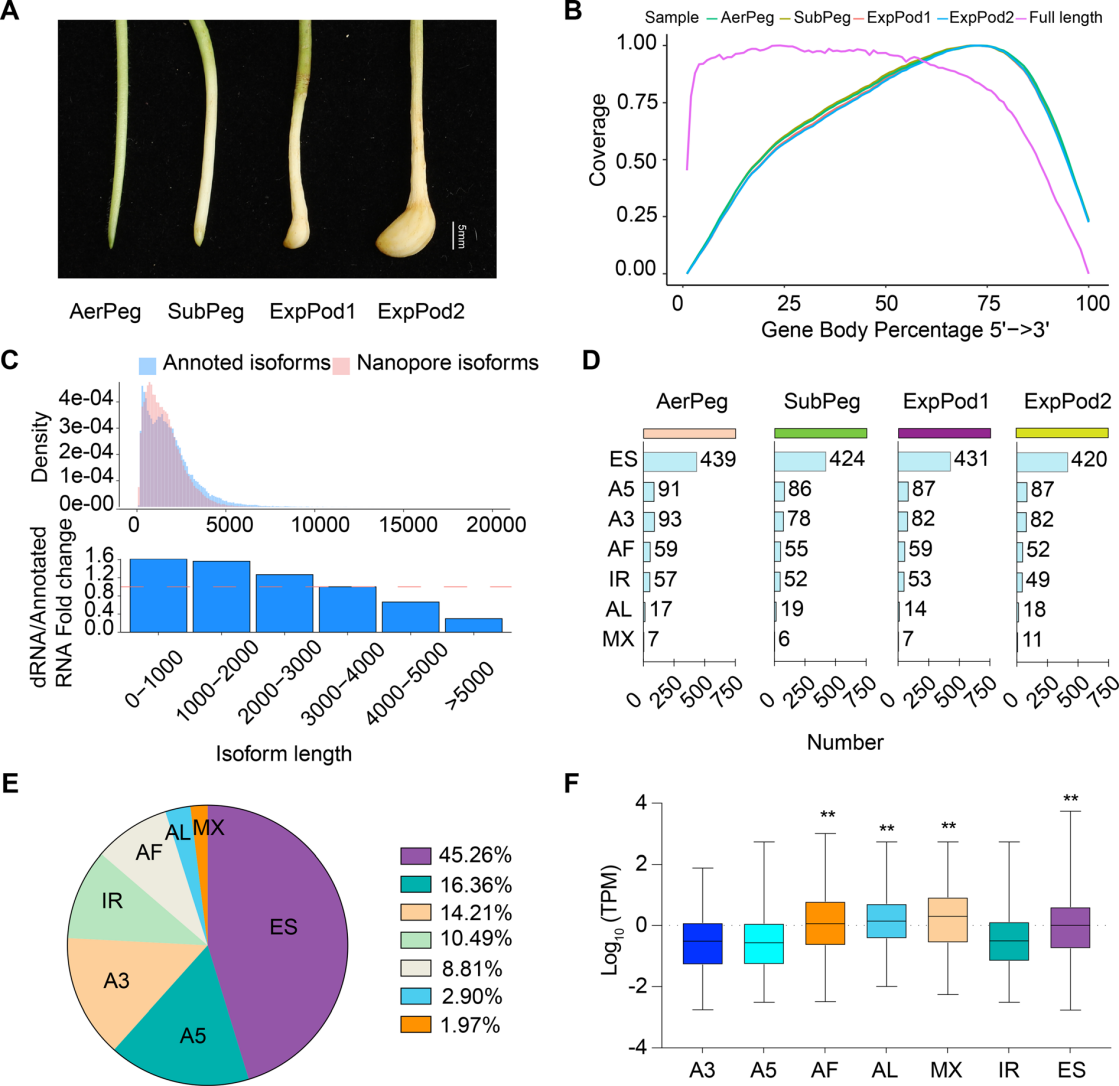
Characterization of alternative splicing (AS) events and full-length RNA isoforms.** A** Phenotypes of peanut pods across four stages of development; bar, 5 mm. **B** Coverage of the Nanopore DRS data. **C** Density distribution (top) and fold change in transcript lengths (bottom) of DRS isoforms and annotated isoforms, with the dashed line denoting a fold change of 1. **D** Number of AS events at different developmental stages. **E** Pie chart showing the proportion of AS events. **F** Boxplots illustrating the relationship between different AS events and expression levels. The box limits represent the 25th and 75th percentiles; center line, 50th percentiles. Whiskers, min and max. Statistical significance was evaluated using a Student’s *t* test and one-way ANOVA (** *P* < 0.01, * *P* < 0.05)

We performed DRS using peanut pods at four developmental stages: AerPeg (Aerial peg: aerial gynophore), SubPeg (Subterranean peg: gynophore having penetrated the soil for 2–3 days without expansion), ExpPod1 (Expanded pod1: gynophore having penetrated the soil for 4–5 days and beginning to expand), and ExpPod2 (Expanded pod2: gynophore having penetrated the soil for 8–10 days and formed a young pod) (Fig. 1A). The filtered reads were subjected to third-generation sequencing. We obtained 70.43 million long reads from 12 samples across the four stages of development, comprising over 5.87 million reads at each stage. The average length of these reads was between 890 nt and 1136 nt, far exceeding the 200-nt read length commonly observed in NGS. We mapped the reads to the peanut genome (https://dev.peanutbase.org), with a mapping rate of up to 98% (Supplemental Table S1). The coverage of full-length reads remained relatively uniform across the transcripts, but short reads were concentrated at the 3′ ends (Fig. 1B). Transcripts of < 3000 bp (as measured by DRS) were notably more abundant than those annotated in the peanut genome (Fig. 1C).

### DRS reveals previously uncharacterized genes and transcripts associated with peanut pod development

**Fig. 2:**
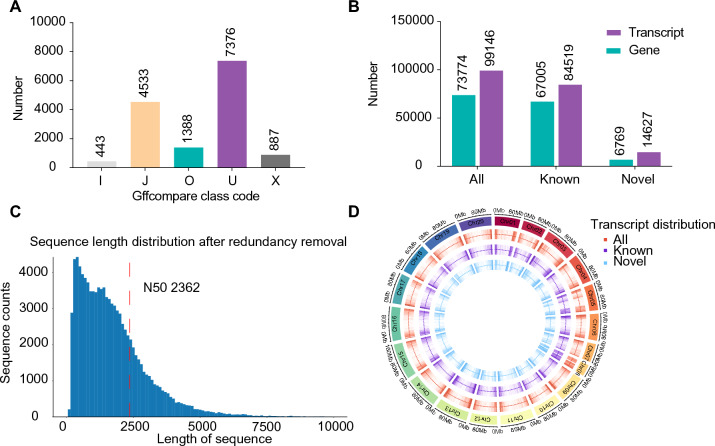
DRS reveals newly identified genes and transcripts in peanuts.** A** Distribution of the types of newly identified transcripts in peanut pods. **B** Numbers of newly identified transcripts and genes. **C** Distribution of CDS lengths of the newly identified transcripts. The *x*-axis represents the length of reads, and the *y*-axis represents the number of reads within the length range; the red dotted line indicates the N50 length. **D** Density distribution map of transcripts on the peanut chromosomes. From outside to inside, chromosomes, transcripts, known transcripts, and newly identified transcripts

We identified previously uncharacterized transcripts and genes by comparing the transcripts sequenced using third-generation DRS in peanut pods with known transcripts from the peanut genome. We divided the transcript regions into five types: O represents a region that overlaps with a region on the same strand in an exon in the reference genome; J represents multiple exons with at least one match; X represents an overlapping exon on the anti-strand; I represents an intron completely contained within the reference transcript; and U represents an unknown transcript. We identified 14,627 previously uncharacterized transcripts, among which the U and J transcripts were represented by 7376 and 4533 members, respectively. The three other types, O, X, and I, comprised 1,388, 887, and 443 transcripts, respectively (Fig. 2A). In total, we identified 6769 previously uncharacterized genes (Fig. 2B).

We predicted the coding sequences (CDS) of all newly discovered transcripts. The CDS lengths of most newly discovered transcripts were between 0 and 5000, and the N50 was 2362 (Fig. 2C). These transcripts were evenly distributed at both ends of the chromosomes in the reference genome (Fig. 2D). Gene Ontology (GO) enrichment analysis of these newly identified transcripts and genes revealed an enrichment in the GO terms regulation of DNA endoreduplication, rejection of self-pollen, defense response to another organism, post-embryonic development, response to auxin, negative regulation of cell population proliferation, and pigment biosynthetic process (Supplemental Fig. S1A).

In addition to protein-coding RNAs, we predicted long non-coding RNAs (lncRNAs) in peanut pods at four stages of development, identifying 6,451 lncRNAs (Supplemental Fig. S2A). These comprised 3,279 lncRNAs (50.83%), 1,113 antisense lncRNAs (17.25%), 493 intronic lncRNAs (7.64%), and 1,566 sense lncRNAs (24.28%) (Supplemental Fig. S2B).

### Transcript abundance varies across four stages of pod development

**Fig. 3:**
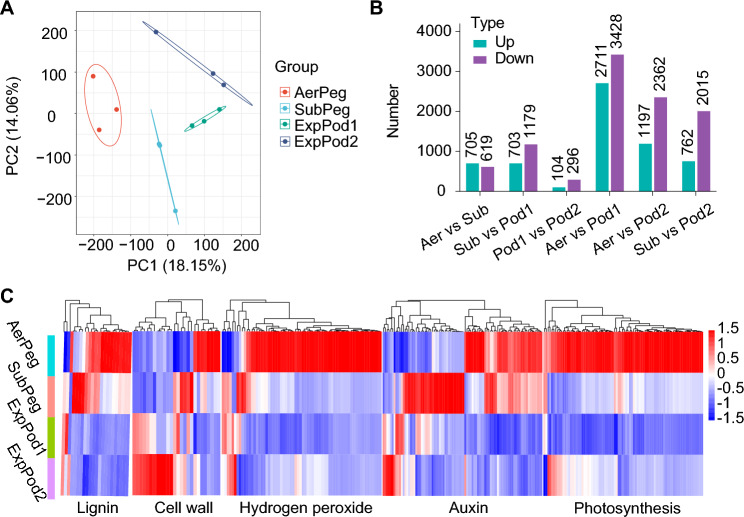
Transcript abundance in peanut pods varies across developmental stages. **A** PCA of transcript levels in peanut pods across four stages of development. **B** Numbers of differentially abundant transcripts. **C** Expression of genes associated with various enriched GO terms

We compared the transcripts in peanut pods at four stages of development using principal component analysis (PCA). The transcript levels were significantly different across the four stages (Fig. 3A and Supplemental Table S2. The greatest number of differentially abundant transcripts was identified between AerPeg and ExpPod1, with 6,139 differentially abundant transcripts (2,711 upregulated and 3,428 downregulated), followed by 3,559 differentially abundant transcripts (1,197 upregulated and 2,362 downregulated) between AerPeg and ExpPod2. Meanwhile, there were fewer differentially abundant transcripts (104 upregulated and 296 downregulated) between ExpPod1 and ExpPod2, indicating that the transcriptional states of these two stages are more consistent (Fig. 3B).

GO enrichment analysis of all pairwise comparisons of differentially abundant transcripts indicated that these transcripts were enriched in photosynthesis, response to oxidative stress, response to auxin, plant-type cell wall organization, and lignin catabolism (Supplemental Fig. S1B). These results suggest that the proteins associated with these functions are more active during peanut pod development and are critical for this process. Photosynthesis-related genes were expressed at the highest levels during the AerPeg stage and at low levels across other developmental stages (Fig. 3C). Genes involved in the response to oxidative stress were expressed at high levels in the AerPeg and SubPeg states and at low levels in the ExpPod1 and ExpPod2 stages (Fig. 3C). These genes were also differentially expressed in response to various other stresses, such as mechanical stress and light/dark stress. Similarly, lignin catabolism-related genes were significantly downregulated during pod development, and genes encoding laccases, which regulate cell size and cell elongation during pod development, showed a similar expression pattern (Fig. 3C). The expression of auxin response factor genes was higher at the AerPeg stage than at the other three stages. Expansin genes were highly expressed throughout pod development, indicating that expansin family members play roles at different stages of peanut pod development (Fig. 3C). The qRT-PCR results and transcriptome analysis results were consistent. The expression of the lignin catabolism-related genes *AhLaccase5*, *AhLaccase10*, and *AhLaccase17* gradually decreased during pod development. The expression of the expansin gene *AhEXP2*, which is related to plant growth and development, gradually increased during pod development. *AhARG7_1*, *AhARG7_2*, and *AhARG7_3* (auxin-responsive gene family members) were expressed at the highest levels prior to peg penetration. In addition, *AhPOD1* was expressed at the highest level during the SubPeg stage (Supplemental Fig. S3). 

To investigate the effects of mechanical stress, light, and other stresses on peanut pod development, we analyzed the transcriptomes of pods at two stages of development: AerPeg (before soil penetration) and SubPeg (after soil penetration). In total, 705 genes were upregulated and 619 genes were downregulated in AerPeg compared to SubPeg (Supplemental Fig. S4A). The major GO terms related to mechanical stress were response to oxidative stress, signaling receptor activity, and the abscisic acid-activated signaling pathway (Supplemental Fig. S4B). Heat map analysis showed that most of the genes associated with these three terms were upregulated during the SubPeg stage compared with the AerPeg stage (Supplemental Fig. S4C). Genes associated with oxidative stress were also differentially expressed across the different stages of pod development.

### Alternative splicing events during peanut pod development

We analyzed alternative splicing (AS) in peanut pods at the four stages of development and identified exon skipping (ES), alternative 5′ splice site (A5), alternative 3′ splice site (A3), and intron retention (IR) events. These AS events were much more frequent at the AerPeg stage than at the three other stages (Supplemental Table S3 and Fig. 1D), with ES making up 45.26% and A5 making up 16.36% of AS at this stage (Fig. 1E), and most AS events representing only one type. However, a mutually exclusive exon (MX) type of AS, in which isoforms contain one exon or another, but never both, was always accompanied by other AS events (Supplemental Fig. S5). We also investigated the relationship between AS and gene expression. There were significantly more transcripts with an alternative first exon (AF), an alternative last exon (AL), MX, and ES types of AS than with the other types of AS (Fig. 1F).

### 3′ UTR length changes during pod development

**Fig. 4:**
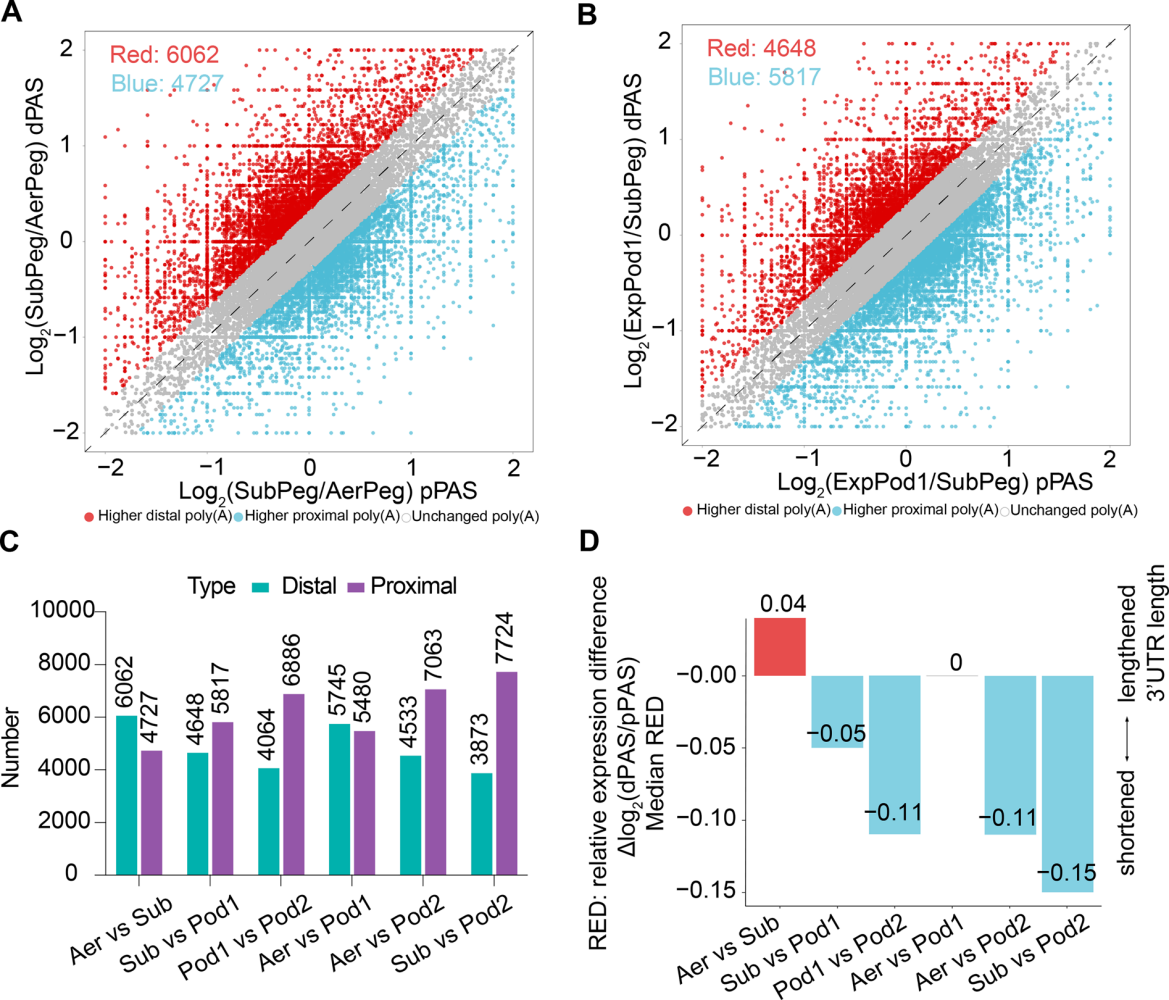
Analysis of APA sites in peanut pods at four stages of development.** A** Scatter plot illustrating the variation in the use of proximal APA sites (pPAS; *x*-axis) and distal polyadenylation sites (dPAS; *y*-axis) through comparisons between SubPeg and AerPeg. **B** Scatter plot illustrating the variation in pPAS (*x*-axis) and dPAS (*y*-axis) usage through comparisons between ExpPod1 and SubPeg. **C** Numbers of differential APA sites. **D** Mean relative expression difference (RED) values obtained by pairwise comparisons. Negative values denote a reduction in global 3′ UTR length, and positive values correspond to an increase in global 3′ UTR length

To examine changes in APA sites during different stages of pod development, we conducted a pairwise comparison of the use of proximal and distal APA sites (Supplemental Table S4). We identified the number of genes that used distal APA sites as well as those that used proximal APA sites (Fig. 4A, B and Supplementary Fig S6). Across the four stages of peanut pod development, the transcripts tended to change their usage from distal APA sites to proximal APA sites (Fig. 4C). To explore the changes in the ratios of distal polyadenylation sites (dPAS) vs. proximal polyadenylation sites (pPAS) in the pairwise comparisons, we quantified global changes in APA sites by calculating relative expression difference (RED) scores. We obtained a maximum RED score of –0.15 between SubPeg and ExpPod2 (Fig. 4D). For all pairwise comparisons, the transcripts with differential APA site usage were primarily enriched in protein folding, mRNA splicing via spliceosome, cell wall organization pectin biosynthetic process, glutamine metabolic process, cell differentiation, and other biological processes (Supplemental Fig. S1C).

### PAL changes during pod development

**Fig. 5:**
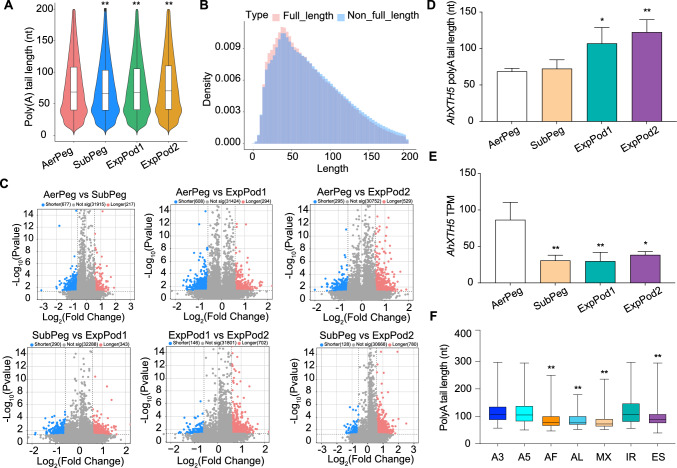
Poly(A) length changes in peanut pods across four stages of development.** A** Changes in poly(A) length in peanut pods across four stages of development. The Mann–Whitney *U* test was used for statistical analysis (** P < 0.01). The box limits represent the 25th and 75th percentiles; center line, 50th percentiles. **B** Poly(A) density distribution map for peanut pods at four stages of development. **C** Volcano plots showing the different PALs in pairwise comparisons between peanut pods at four stages of development. **D** Poly(A) tail length of *AhXTH5* analyzed by one-way ANOVA (**P < 0.01, * P < 0.05). **E** Analysis of *AhXTH5* expression levels by one-way ANOVA (**P < 0.01, * P < 0.05). **F** Boxplots showing the relationships between different AS events and PALs analyzed using one-way ANOVA (** *P* < 0.01, * P < 0.05). The box limits represent the 25th and 75th percentiles; center line, 50th percentiles. Whiskers, min and max

To examine changes in PAL in peanut pods during different stages of development, we evaluated the entire lengths of poly(A) sequences in each sample (Supplemental Table S5). The median length of poly(A) tails was 84.38 nt at the AerPeg stage (before the gynophore is buried). The median length of poly(A) tails was only 79.91 nt at the SubPeg stage and 81.6 nt at the ExpPod1 stage (after the gynophore is buried). The length of poly(A) tails was significantly longer at the ExpPod2 stage (when the gynophore develops into a young pod). The median length of poly(A) tails was 86.59 nt at the ExpPod2 stage. We detected dynamic changes in PAL during different stages of pod development (Fig. 5A). We investigated the relationship between poly(A) length and transcript stability. The median PAL for full_length reads was shorter than the median PAL for non_full_length reads (Fig. 5B).

We performed pairwise comparisons of poly(A) tails in pods at different stages of development. We identified that 702 (ExpPod1 vs. ExpPod2), 343 (SubPeg vs. ExpPod1), 780 (SubPeg vs. ExpPod2), 294 (AerPeg vs. ExpPod1), 217 (AerPeg vs. SubPeg), and 529 (AerPeg vs. ExpPod2) transcripts had longer poly(A) tails. Among these, 146 (ExpPod1 vs. ExpPod2), 290 (SubPeg vs. ExpPod1), 128 (SubPeg vs. ExpPod2), 608 (AerPeg vs. ExpPod1), 677 (AerPeg vs. SubPeg), and 295 (AerPeg vs, ExpPod2) transcripts had shorter poly(A) tails (Fig. 5C). GO enrichment analysis revealed that genes responsible for changes in PAL during peanut pod development were primarily enriched in phosphatidylglycerol biosynthesis, tRNA wobble uridine modification, response to desiccation, phospholipid transport, microtubule cytoskeletal organization, chorismate biosynthesis process, rhythmic process, nitrate assimilation, ethylene activation of signaling pathways, xylose metabolism, and xylan catabolism, among others (Supplemental Fig. S1D). Correlation analysis (Supplemental Fig. S7A) suggested a negative correlation between PAL in transcripts and gene expression levels. For instance, the PAL of *AhXTH5*, a gene involved in xyloglucan metabolism, became longer during peanut pod development (Fig. 5D); however, this gene was downregulated during pod development (Fig. 5E). In addition, an analysis of the relationship between variable AS and PAL indicated that the poly(A) sequences in transcripts exhibiting AF, AL, ES, and MX were shorter than those of the other types (Fig. 5F).

### m^6^A modification of RNAs varies during pod development

**Fig. 6:**
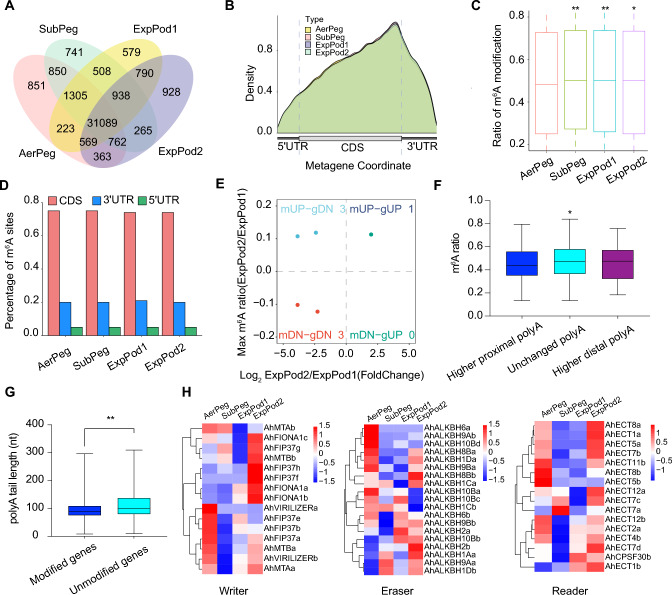
Analysis of m^6^A methylation in peanut pods at four stages of development. **A** Venn diagram showing the number of genes with m^6^A modification. **B** Density distribution of m^6^A sites along the gene body. **C** Boxplot showing the rates of m^6^A modification. The Mann–Whitney *U* test was employed for statistical analysis (** P < 0.01, * P < 0.05). The box limits represent the 25th and 75th percentiles; center line, 50th percentiles. Whiskers are 1.5 × interquartile range extending from the edge of the box. **D** Percentage of m^6^A modification sites within coding sequences (CDS) and untranslated regions (UTRs). **E** Scatter plot of the results of pairwise comparisons of ExpPod2 versus ExpPod1 showing the relationships of m^6^A ratio with gene expression. **F** Boxplot showing the ratio of m^6^A sites with altered proximal or distal usage (ExpPod2 versus ExpPod1). Statistical significance was assessed by Student’s *t* test and one-way ANOVA (* *P* < 0.05). The box limits represent 25th and 75th percentiles; center line, 50th percentiles. Whiskers, min and max. **G** Boxplot showing the PALs of modified and unmodified genes. The unpaired *t* test was used for statistical analysis (** P < 0.01). The box limits represent the 25th and 75th percentiles; center line, 50th percentiles. Whiskers, min and max. **H** The expression levels of genes involved in m^6^A modification in peanut

Based on the DRS library, we analyzed the methylation of m^6^A in pods across different stages of development (Supplemental Table S6). We identified 36,012 (AerPeg), 36,458 (SubPeg), 36,001 (ExpPod1), and 35,704 (ExpPod2) m^6^A sites during peanut pod development (Fig. 6A). The m^6^A modification sites were enriched near the m^6^A stop codon and 3′ UTR (Fig. 6B). The rates of m^6^A modification at different stages were 49% (AerPeg), 50% (SubPeg), 50% (ExpPod1), and 50% (ExpPod2) (Fig. 6C), indicating that the modification rate was slightly lower in AerPeg than at the three other stages. Moreover, most of the m^6^A modifications were located within the CDS (74%–75%), 3′ UTR (20%–21%), and 5′ UTR (5%) (Fig. 6D).

We examined genes with changes in m^6^A methylation and significantly different transcript levels during pod development. We identified 3 (AerPeg vs. SubPeg), 8 (SubPeg vs. ExpPod1), 5 (ExpPod1 vs. ExpPod2), 53 (SubPeg vs. ExpPod2), 41 (AerPeg vs. ExpPod1), and 69 (AerPeg vs. ExpPod2) differentially expressed genes with changes in m^6^A modification levels (Fig. 6E and Supplemental Fig. S8). The rate of m^6^A modification appeared to be lower in regions with higher proximal poly(A) levels compared with regions with higher distal poly(A) and unchanged poly(A) levels (Fig. 6F). The PAL was significantly shorter for modified transcripts compared with unmodified transcripts (Fig. 6G). There was no correlation between the m^6^A modification ratio and gene expression (Supplemental Fig. 7B).

Finally, we examined the expression levels of key genes involved in m^6^A modification. The writer genes *AhFIONA1a*, *AhFIONA1b*, *AhFIONA1c*, *AhFIP37g*, *AhFIP37h*, *AhFIP37f*, and *AhMTBb* were significantly upregulated during the ExpPod2 stage (Fig. 6H). In particular, the expression of *AhFIONA1a* and *AhFIONA1b* continued to increase gradually throughout development, following very similar patterns. *AhMTAa*, *AhVIRILIZERa*, *AhVIRILIZERb*, and *AhMTBa* were abundantly expressed at the AerPeg stage; these genes function in the entrance of the peg into the soil. The eraser genes *AhALKBH2a* and *AhALKBH10Bb* were highly expressed at the ExpPod1 and ExpPod2 stages. Similarly, most reader genes were abundantly expressed during the AerPeg and ExpPod2 stages (Fig. 6H).

## Discussion

Peanut pod development is essential for the yield and quality of peanuts. Many studies have examined transcription during peanut pod development (Li et al. 2017; Liu et al. 2022; Wan et al. 2017); however, analysis at the transcriptional level alone is insufficient to completely characterize gene function. The regulation of gene expression is significantly influenced by post-transcriptional changes such as AS, poly(A) tailing, APA, and m^6^A methylation. However, the constraints of RNA-Seq technology limit the detailed characterization of these post-transcriptional processes. DRS can simultaneously identify previously uncharacterized genes, transcripts, poly(A) tails, APA, and m^6^A modifications (Li et al. 2023; Parker et al. 2020). However, to date, no studies on post-transcriptional changes during peanut pod development have been reported. In this study, we identified 6769 previously uncharacterized genes and 14,627 newly discovered transcripts in developing peanut pods via DRS. The identification of these genes and transcripts will facilitate the annotation of the peanut genome. The newly discovered transcripts were predominantly enriched in post-embryonic developmental processes, making them promising targets of further analysis.

Auxin is essential for the formation of peanut pods (Cui et al. 2022b; Lv et al. 2023). A series of auxin-related genes (*AUX28*, *IAA9*, *YAB5*, and *ARF*) involved in peanut pod and seed development have been identified (Cui et al. 2024). In this study, we identified numerous auxin-responsive genes that were highly expressed during the AerPeg and SubPeg stages of pod development. Laccases are involved in cell development and affect plant growth, as demonstrated in various plants (Berthet et al. 2011; Blaschek et al. 2022). The size of peanut pods is negatively correlated with lignin content (Lv et al. 2022), which is consistent with our finding that numerous laccase genes associated with lignin metabolism are significantly downregulated from the peg penetration to pod expansion stages. The maize (*Zea mays*) *zmexpb12* mutant contains smaller seeds and smaller cells than the wild type (Ji et al. 2022). We determined that expansin genes were highly expressed during all four stages of peanut pod development, with each gene likely serving specific functions. These genes provide a theoretical basis for subsequent studies of peanut pod development. 

The poly(A) tail is essential for exporting most mature mRNAs from the nucleus and influences mRNA stability (Fuke and Ohno 2008). We determined that PAL is negatively correlated with gene expression levels, which is consistent with a previous report in *Arabidopsis* (Parker et al. 2020). Similarly, we observed higher rates of m^6^A modification during the SubPeg and ExpPod1 stages, whereas the PAL was shorter at these two stages. This result is consistent with our finding that the PALs of m^6^A-modified genes are significantly longer than those of unmodified genes.

Transcripts shifted from the utilization of distal APA sites to the greater utilization of proximal APA sites across the four stages of peanut pod development. In *Arabidopsis*, numerous transcripts associated with nitrate signaling in the *cpsf30-2* mutant shift toward the APA site associated with the m^6^A peak, suggesting that these m^6^A-bearing transcripts are regulated by APA (Hou et al. 2021). In peanut, the rate of m^6^A modification appears to be lower in regions with higher proximal poly(A) ratios than in regions with higher distal poly(A) and unchanged poly(A) ratios (Fig. 6F). In addition, an analysis of the relationship between variable AS and PAL indicated that the length of poly(A) tails in transcripts with AF-, AL-, and MX-type AS is shorter than those displaying the other types (Fig. 5F). Genes with altered APA sites are enriched in cell wall organization, pectin biosynthetic process, cell differentiation, and other biological processes during peanut pod development. These results shed light on the changes in post-transcriptional modifications during peanut pod development and lay a theoretical foundation for further study of the roles of these modifications in peanut pods.

m^6^A functions in various processes during plant growth and development, including embryogenesis (Zhong et al. 2008), the floral transition (Wang et al. 2022), root development (Zhang et al. 2022), leaf morphology (Arribas-Hernandez et al. 2018), and fruit ripening (Zhou et al. 2021, 2019). m^6^A is the most abundant internal mRNA modification regulated by writer, eraser, and reader proteins (Tang et al. 2023). The distribution of m^6^A in specific transcript regions and conserved motifs identified in this study are similar to those reported previously; these modifications can be integrated into m^6^A RRACH (R = A/G; H = A/C/U) motifs, which are enriched near 3′ UTRs and stop codons (Anderson et al. 2018; Parker et al. 2020). Poly(A) tails are significantly shorter in modified vs. unmodified transcripts (Fig. 6G).

Finally, we examined the expression levels of genes responsible for m^6^A modification in peanut. Most of these genes were differentially expressed during pod development. For example, *AhALKBH2a* and *AhALKBH10Bb* were highly expressed at the ExpPod1 and ExpPod2 stages. Similarly, most reader genes were abundantly expressed during the AerPeg and ExpPod2 stages. The *Arabidopsis alkb10B* mutant shows increased methylation of *FLOWERING LOCUS T* mRNA and decreased mRNA stability, delaying flowering (Duan et al. 2017). Analysis of these key m^6^A modifiers offers a theoretical basis for further studying their functions in peanut pod development.

In this study, we identified complex RNA modifications during peanut pod development. Specifically, the mean length of full-length poly(A) tails was lower during the SubPeg and ExpPod1 stages than during the other developmental stages. There was a negative relationship between poly(A) length and gene expression. The distal APA sites in transcripts were more widely utilized during the SubPeg stage, whereas the proximal APA sites in transcripts were employed more frequently during the other developmental stages. There was no correlation between m^6^A modification and gene expression in peanut. We also examined the expression of genes related to m^6^A modification during pod development. Our findings provide a theoretical basis for further investigating the functions of RNA modification in peanut pod development. The discovery of previously uncharacterized genes and transcripts in peanut will facilitate further study of their functions in pod development.

## Materials and methods

### Plant material

*Arachis hypogaea* cv. Tifrunner (Clevenger et al. 2016) plants were grown for 40 days in a greenhouse at the Institute of Advanced Agricultural Sciences, Peking University, Weifang, China. Pegs and pods were harvested from 30 plants at four distinct growth stages: AerPeg (Aerial peg: aerial gynophore), SubPeg (Subterranean peg: gynophore has penetrated the soil for 2–3 days without expansion), ExpPod1 (Expanded pod 1: gynophore has penetrated the soil for 4–5 days and begun to expand), and ExpPod2 (Expanded pod 2: gynophore has penetrated the soil for 8–10 days and formed a young pod). Total RNA was isolated from the samples using a Plant RNA Extraction Kit (Omega R6827-01 China). Each sample contained 20 μg total RNA for DRS. The RNA was cleaned and concentrated using a NEBNext Poly(A) mRNA Magnetic Isolation Module (E7490S) according to the manufacturer’s instructions. From each sample, 1 μg of total RNA was used to examine RNA integrity and concentration using a Nanodrop spectrophotometer (Thermo Fisher Scientific). The *Arachis_hypogaea*_J5K5.main_1.4K0L_peanutbase genome was used for bioinformatics analysis (https://dev.peanutbase.org).

### Nanopore sequencing library construction and sequencing

RNA from each sample was used for DRS library preparation following the Oxford Nanopore DRS protocol (SQK-RNA002, Oxford Nanopore Technologies). For the reversed connector connection, a 15-μL reaction was generated by mixing 9 μL prepared RNA, 3 μL NEBNext Quick Ligation Reaction Buffer (NEB), 1 μL RT Adapter (RTA; SQK-RNA002), and 2 μL T4 DNA Ligase (NEB) and incubated at 25 °C for 10 min. After adding 8 μL 5 × first-strand buffer (NEB), 2 μL 10 mM dNTPs (NEB), 9 μL nuclease-free water, 4 μL 0.1 M DTT (Thermo Fisher Scientific), and 2 μL SuperScript III Reverse Transcriptase (Thermo Fisher Scientific, 18,080,044) to the 15 μL reaction system, the sample was incubated at 50 °C for 50 min, followed by 70 °C for 10 min. The reverse-transcribed mRNA was purified using 1.8 Agencourt RNAClean XP beads (BECKMAN, A63987) and washed with 23 μL nuclease-free water. Sequencing adapters were added to the mRNA using 8 μL NEBNext Quick Ligation Reaction Buffer, 6 μL RNA Adapter (RMX), and 3 μL T4 DNA Ligase. The mixture was purified and washed again as described above, and 50 μL RRB (SQK-RNA002) was added to the sample, along with 35 μL nuclease-free water. This final reaction system was loaded into a Nanopore R9.4 sequencing microarray and sequenced for 48–72 h using a PromethION sequencer (Oxford Nanopore Technologies).

### Nanopore DRS and bioinformatics analysis

Nanopore DRS sequencing produces unprocessed raw sequencing signals in FAST5 format. Base calling was performed using Guppy software (version: 5.0.16; Oxford Nanopore Technologies) to convert the data from FAST5 to FASTQ format. The raw reads were filtered using NanoFilt (version: 2.8.0; options: -q 7 -l 50) to acquire valid data (De Coster et al. 2018). SeqKit (version: 0.12.0; options: default) was used for statistical analysis (Shen et al. 2016b). The filtered DRS reads were corrected using fmlrc2 (version: 0.1.4; options: default) based on the second-generation Illumina sequencing data (Wang et al. 2018). The error-corrected transcript sequences were aligned to the reference genes using minimap2 software (version: 2.17-r941; options: -ax splice-uf-k14) (Li 2018). Fair software (version: 1.5.0; options: -t 20) was used to obtain consistent sequences based on the alignment results (Tang et al. 2020). To reduce the redundancy of the results, alignments with only differences in exons at the 5′ end were merged. Non-redundant transcript sequences were obtained using StringTie software (version: 2.1.4; options: -conservative-L-R) (Pertea et al. 2015). Gffcompare software (version: 0.12.1; options: -R-C-K-M) was used to compare the transcripts with known transcripts in the genome and to identify novel transcripts and genes (Pertea and Pertea 2020).

### Structural analysis of gene transcripts

Each transcript was compared with a known transcript in the genome. If a region outside the boundary of the original transcript was detected, the UTR of the transcript was extended upstream and downstream to correct the transcript boundary. SUPPA2 software (https://github.com/comprna/SUPPA; options: -f IOE -e se SS MX RI FL) was used to obtain the alternative splicing type of each sample, and SUPPA2 (DiffSplice) was used to identify the differences in alternative splicing between groups. LncRNAs are a class of RNAs that do not code for proteins and have transcripts exceeding 200 nt in length. CNCI (version:2.0; default settings) (Sun et al. 2013), CPC2 (standalone version, Python 3 v1.0) (Kong et al. 2007), and PLEK (Li et al. 2014) were used to predict the coding potentials of newly identified transcripts fitting this description. Tapas software (https://github.com/arefeen/TAPAS Parameter: -L 50) was used to identify APA sites in combination with the reference sequence from the RefSeq database.

### Differential expression analysis of DRS transcripts

The DRS consensus reads were aligned to novel reference transcripts using minimap2 (version: 2.17 options: -a -k14 -uf -x splice -secondary = no) (Li 2018). To standardize the estimated gene/transcript expression levels between transcripts and experiments, the expression levels were quantified using transcripts per kilobase million (TPM). Transcript expression levels were measured using Salmon software (version 1.4.0) (Patro et al. 2017). Differential expression analysis was conducted using the expression level reads count data of the transcripts in each sample. DESeq2 software (version: 1.26.0) was used for differential expression analysis (Love et al. 2014). Differentially expressed transcripts were identified using the thresholds *P* < 0.05 and |log_2_(fold change)|> 1.

### Analysis of poly(A) tail length

Poly(A) tail length in the raw data was examined using NanoPolish (version: 0.13.2) (De Coster et al. 2018). A Mann–Whitney *U*-test was used to test for differences in PAL at the transcriptome level. Transcripts with significant differences in PAL between samples were characterized as follows: fold change = Control_median/Treatment_median; thresholds: fold change > 1.5 and *P*-value < 0.05.

### Analysis of APA sites

The poly(A) insertion sites represent the endpoints at which the sequencing reads were aligned to the reference genome. After the reads were mapped to the reference genome using minimap2, the aligned endpoint positions were isolated from BAM files, and poly(A) sites were identified, clustered, and annotated using Quantifypoly(A) (Ye et al. 2021). To analyze APA sites in 3′ UTRs, the two most abundant poly(A) sites in the final exon were identified and designated as proximal polyadenylation sites (pPAS) and distal polyadenylation sites (dPAS) according to their locations relative to the 3′ end of the coding sequence. A difference in relative abundance of more than 30% (|log_2_[DPAS1/DPAS2] – log_2_[PPAS1/PPAS2]|) represents a significant difference in the length of the 3 UTR.

### Analysis of m^6^A modification

GFFRead software was employed to extract the corresponding transcriptome sequences using the annotation file (Pertea and Pertea 2020). Sequential raw Nanopore signals were assigned to genome-specific locations using Tombo software (Stoiber et al. 2017). The m^6^A sites were identified using the published Nanom6A Pipeline with default parameters (Gao et al. 2021). The m^6^A ratio was calculated using the following formula: number of modified reads/(number of modified reads + number of unmodified reads). Fisher’s exact test was employed to identify differentially expressed m^6^A sites; m^6^A sites with a mean difference in m^6^A ratio for the same site > 0.1 and *P* < 0.05 were defined as differentially expressed m^6^A sites.

### GO enrichment analysis

ClusterProfiler (version 3.14.3) was utilized for GO enrichment analyses (Yu et al. 2012). A false discovery rate (FDR) < 0.05 was established using the Benjamini and Hochberg multiple means test. Enrichment results display only the top ten most significant items. A cluster heatmap of the bioinformatics data was constructed using OECloud (https://cloud.oebiotech.com).

## Supplementary Information

Below is the link to the electronic supplementary material.

Supplementary file1 (DOCX 24694 KB)

Supplementary file2 (XLSX 11 KB)

Supplementary file3 (XLSX 11484 KB)

Supplementary file4 (XLSX 65 KB)

Supplementary file5 (XLSX 8190 KB)

Supplementary file6 (XLSX 6780 KB)

Supplementary file7 (XLSX 41679 KB)

## Data Availability

The raw FAST5 data have been submitted to the Genome Sequence Archive at the National Genomics Data Center (Chen et al. 2021), Beijing Institute of Genomics, Chinese Academy of Sciences/China National Center for Bioinformation (GSA: CRA019397). The NGS data for each sample have been deposited in the Sequence Read Archive (SRA) at the National Center for Biotechnology Information under accession number PRJNA1136993.
