# Supplementary material for: Identification of post-transcriptional regulation reveals complexity in peanut pod development by Direct RNA
Source: aBIOTECH. 2025 Sep 8;6(3):554–68. doi: 10.1007/s42994-025-00224-5 (PMC12454814; doi:10.1007/s42994-025-00224-5)
Supplement: Supplementary file 1 — Supplementary file1 (DOCX 24694 KB) [file 42994_2025_224_MOESM1_ESM.docx]

**Direct RNA sequencing reveals the post-transcriptional regulation of peanut pod development**

Wei Wang^#1^, Haosong Guo^#1,2^, Jianxin Bian^#1^, Fa Cui^2^, Xiaoqin Liu^1^*

^1^ Peking University Institute of Advanced Agricultural Sciences, Shandong Laboratory of Advanced Agriculture Sciences at Weifang, Weifang 261325, China

^2^ College of Agriculture, Ludong University, Yantai 264025, China

# These authors contributed equally to this article.

* Corresponding author: Xiaoqin Liu; Email: xiaoqin.liu@pku-iaas.edu.cn

**This file includes:**

1, Legend of Supplementary Tables

2, Supplementary Figure 1 to 8

**Supplemental Table S1 Direct RNA sequencing data analysis** Note: Sample: sample name; TotalBase: number of bases; TotalReads: number of reads; MaxLen: maximum length of data; AvgLen: average length of data; N50 and L50: all reads are sorted from long to short and then accumulated in turn. When the sum of a sequence is just more than half of the total sequence length, the length of the sequence is N50, and the total number of accumulated sequences is L50; N90 and L90: calculated according to more than 90% of the total sequence, the algorithm is the same as N50 and L50; meanQ: average quality value. Mapped reads is the reads of the reference genome on the alignment; map _ rate is the alignment rate.

**Supplemental Table S2 The list of transcript expression levels TPM**

**Supplemental Table S3 The list of alternative splicing events**

**Supplemental Table S4 The list of alternative polyadenylation sites**

**Supplemental Table S5 The identification of polyA tail length**

**Supplemental Table S6 The m^6^A modifications based on the DRS**

**Supplementary figure**


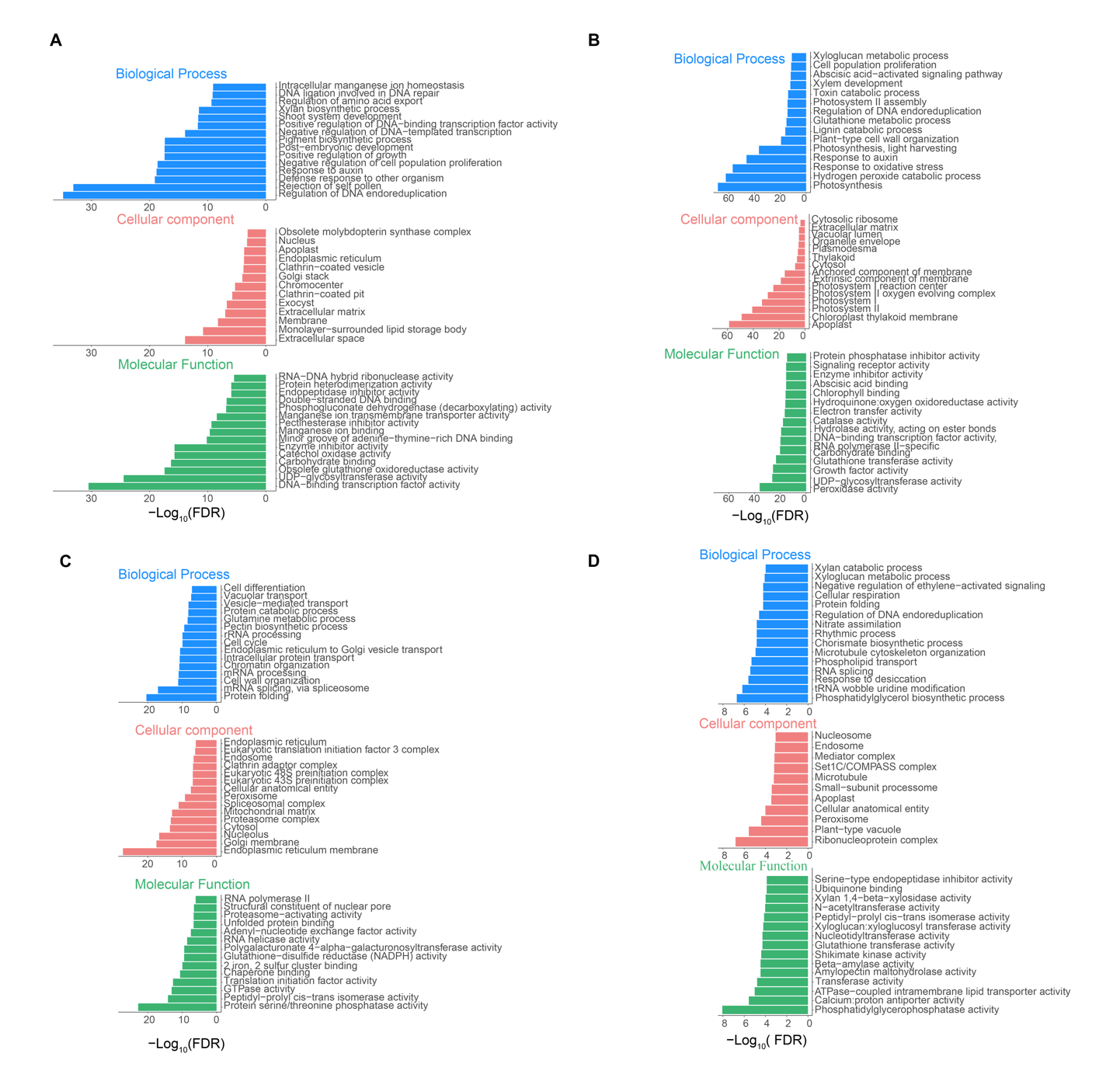


**Supplemental Fig. S1 GO enrichment analysis A** GO enrichment analysis of novel transcripts from peanut pods. **B** GO enrichment analysis of all differential transcripts. **C** GO enrichment analysis of different APA sites. **D** GO enrichment analysis of differential polyA genes across four developmental stages of peanut pods.

**
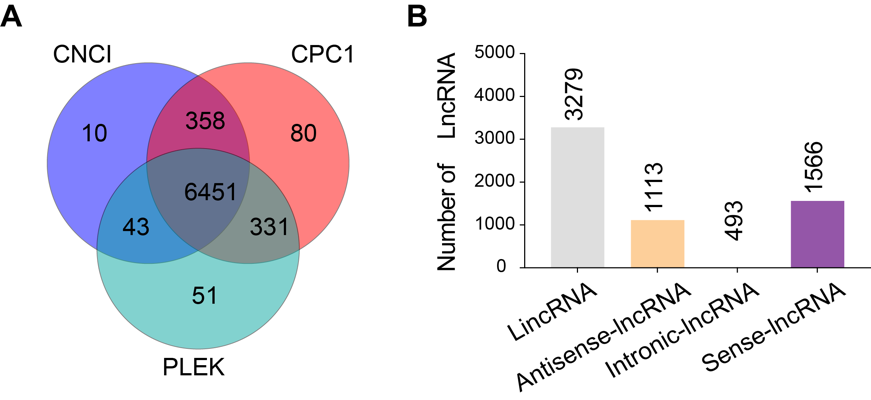
**

**Supplemental Fig. S2 Identification and quantification of LncRNA** **A** Venn diagram showing LncRNA. LncRNA prediction number statistic. LncRNA was predicted by CNCI, CPC2 and PLEK software. **B** Statistics of the number of LncRNAs of different total classes. Intronic lncRNA: Produced primarily in the intron region of the coding gene; Antisense lncRNA: Produced primarily from the antisense strand of the coding gene; sense lncRNA: LncRNA overlapping a protein-coding transcript; LincRNA: LncRNA not overlapping a protein-coding transcript.


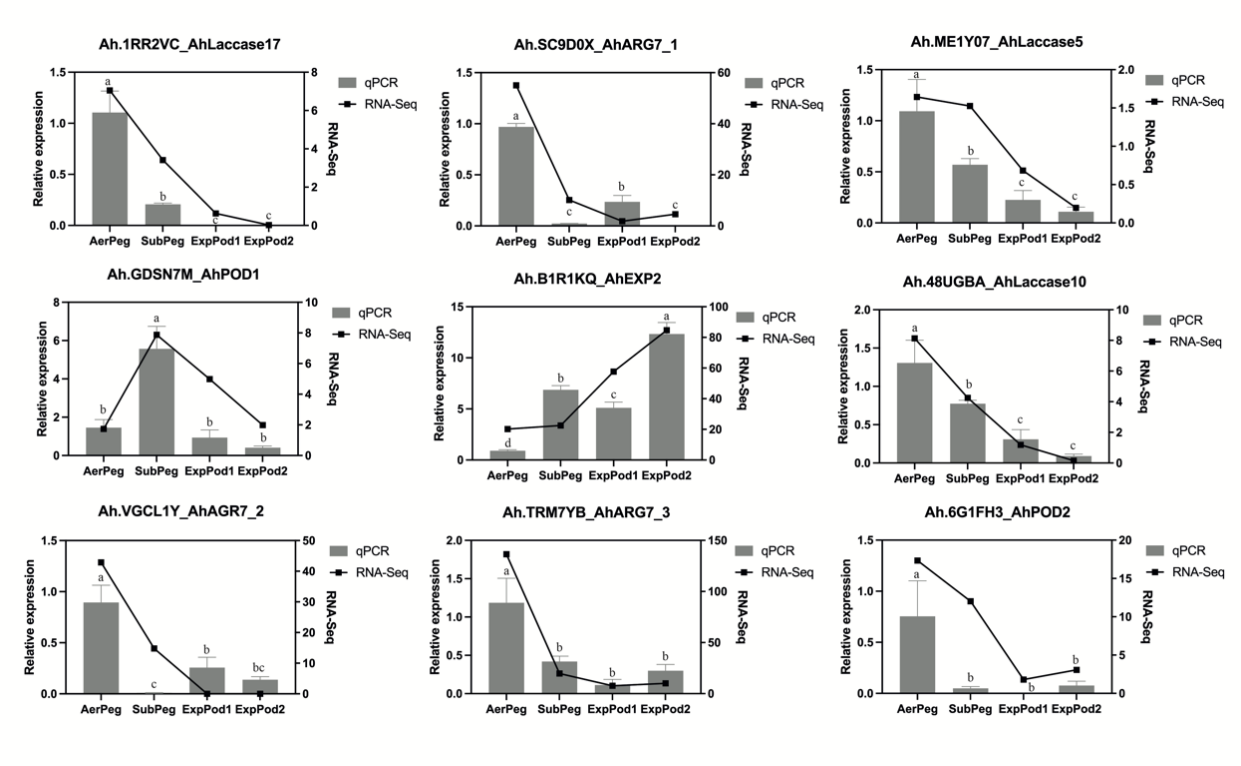


**Fig. S3 qRT-PCR validation of key genes identified in the transcriptome** Validation of the RNA sequencing (RNA-Seq) results by real-time quantitative PCR (qRT-PCR).


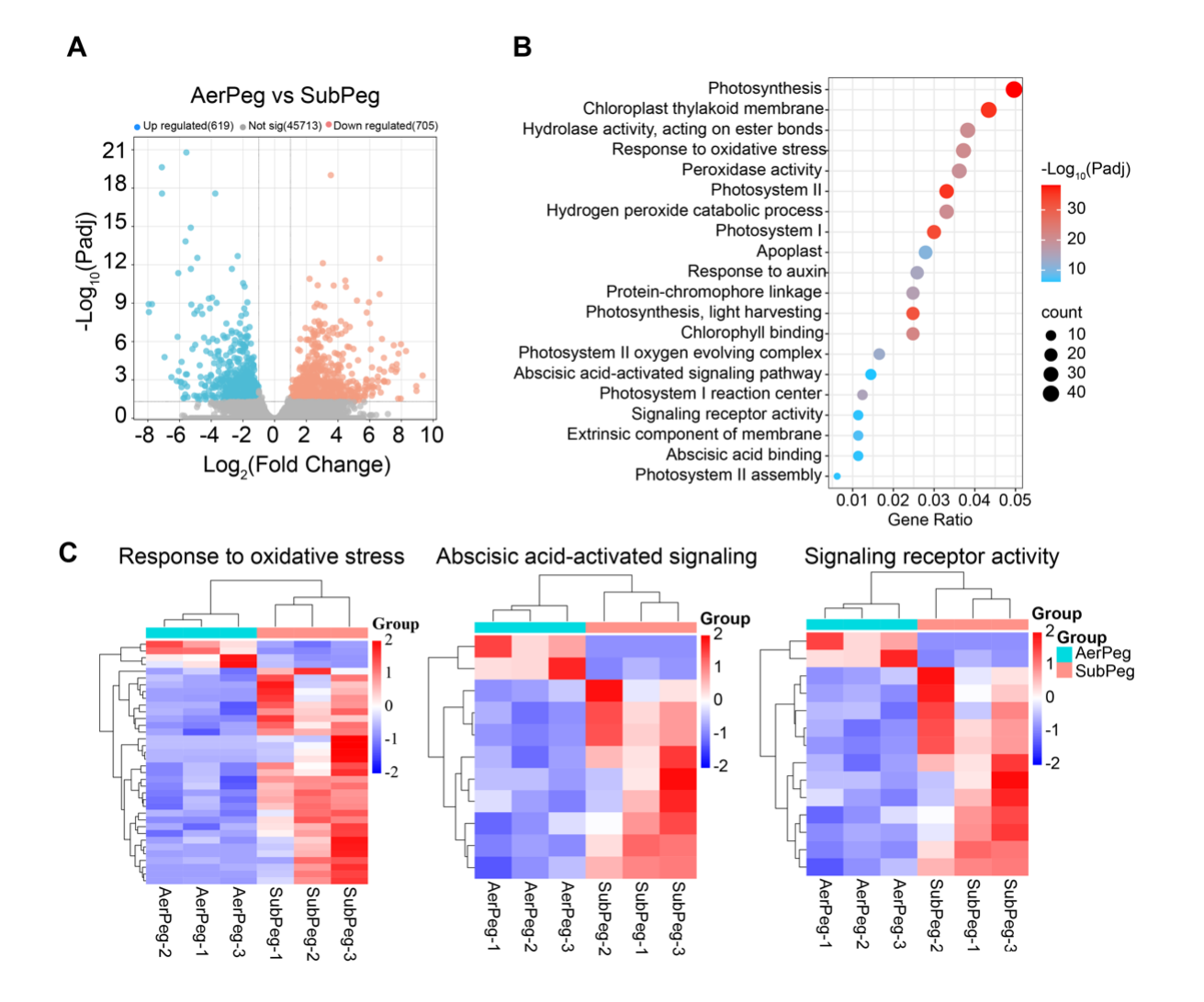


**Fig. S4. Transcriptome analysis of peanut pod development at AerPeg and SubPeg stages A** The volcano map shows the differentially expressed genes between AerPeg and SubPeg. **B** GO enrichment analysis of differential genes. **C** The expression of genes related to GO enrichment pathway.


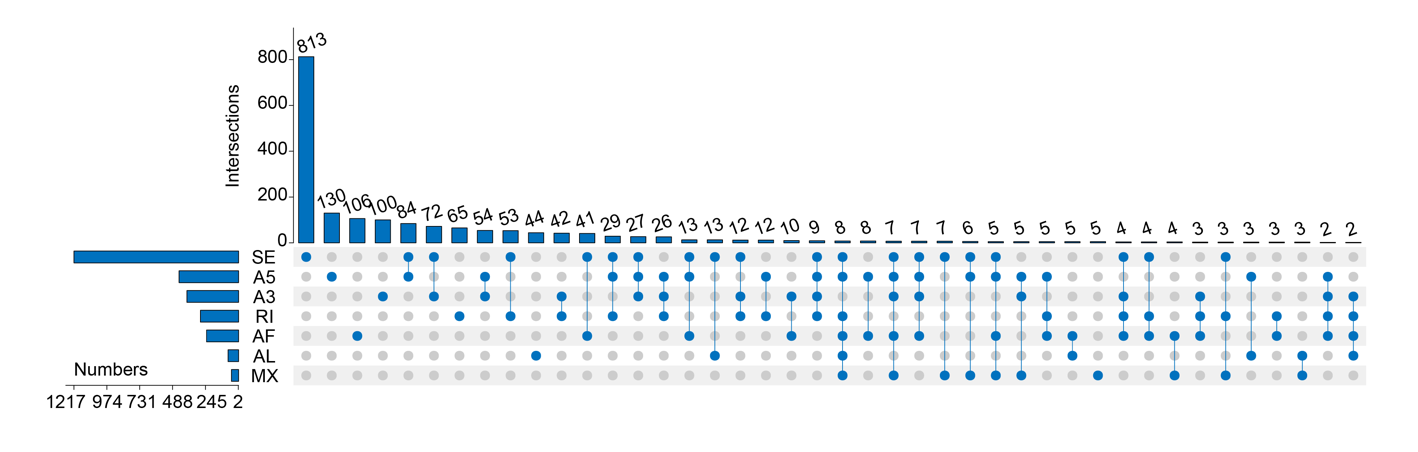


**Supplemental Fig. S5 Number of isomers formed by different AS events.**

SE refers to exon skipping; MX refers to mutually exclusive exons; A5 refers to 5' end alternative splicing; A3 refers to 3' end alternative splicing; RI refers to intron retention; AF refers to the first exon alternative splicing; AL refers to the last exon alternative splicing.

**
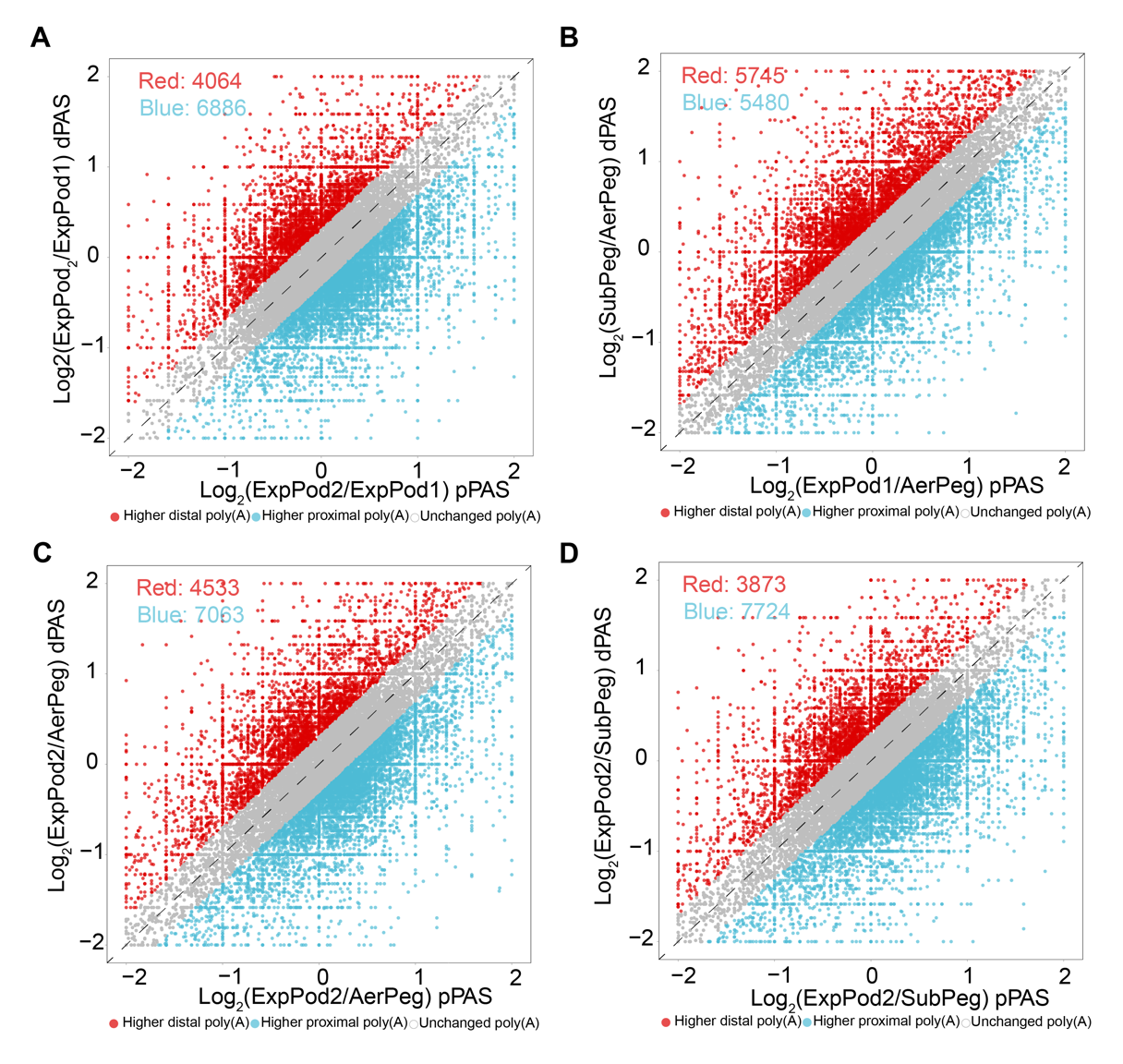
**

**Supplemental Fig. S6 Scatter plot showing the change of pPAS type (X axis) and dPAS type (Y axis) in pairwise comparisons** **A** Scatter plot illustrating the variation of pPAS type (X-axis) and dPAS type (Y-axis) through comparisons between ExpPod2 and ExpPod1. **B** Scatter plot illustrating the variation of pPAS type (X-axis) and dPAS type (Y-axis) through comparisons between ExpPod1 and AerPeg. **C** Scatter plot illustrating the variation of pPAS type (X-axis) and dPAS type (Y-axis) through comparisons between ExpPod2 and AerPeg. **D** Scatter plot illustrating the variation of pPAS type (X-axis) and dPAS type (Y-axis) through comparisons between ExpPod2 and SubPeg.

**
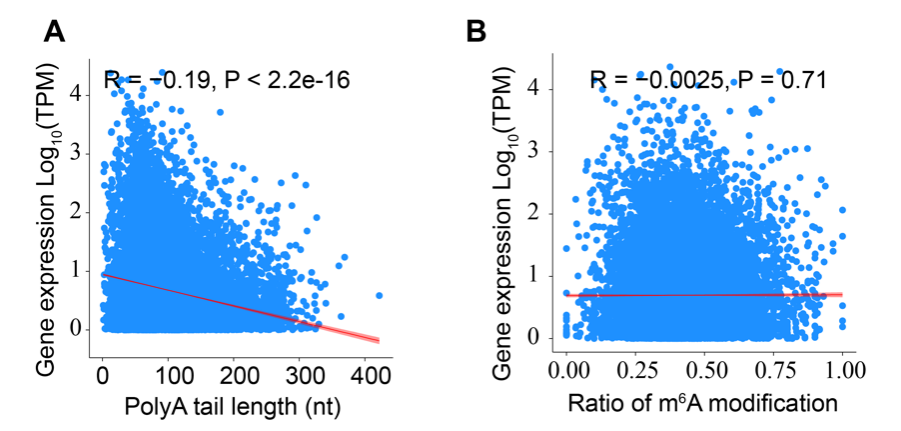
**

**Supplemental Fig. S7 Correlation analysis of m^6^A modification ratio and polyA length with expression level A** Correlation between polyA length and gene expression. **B** Correlation between m^6^A modification and gene expression.


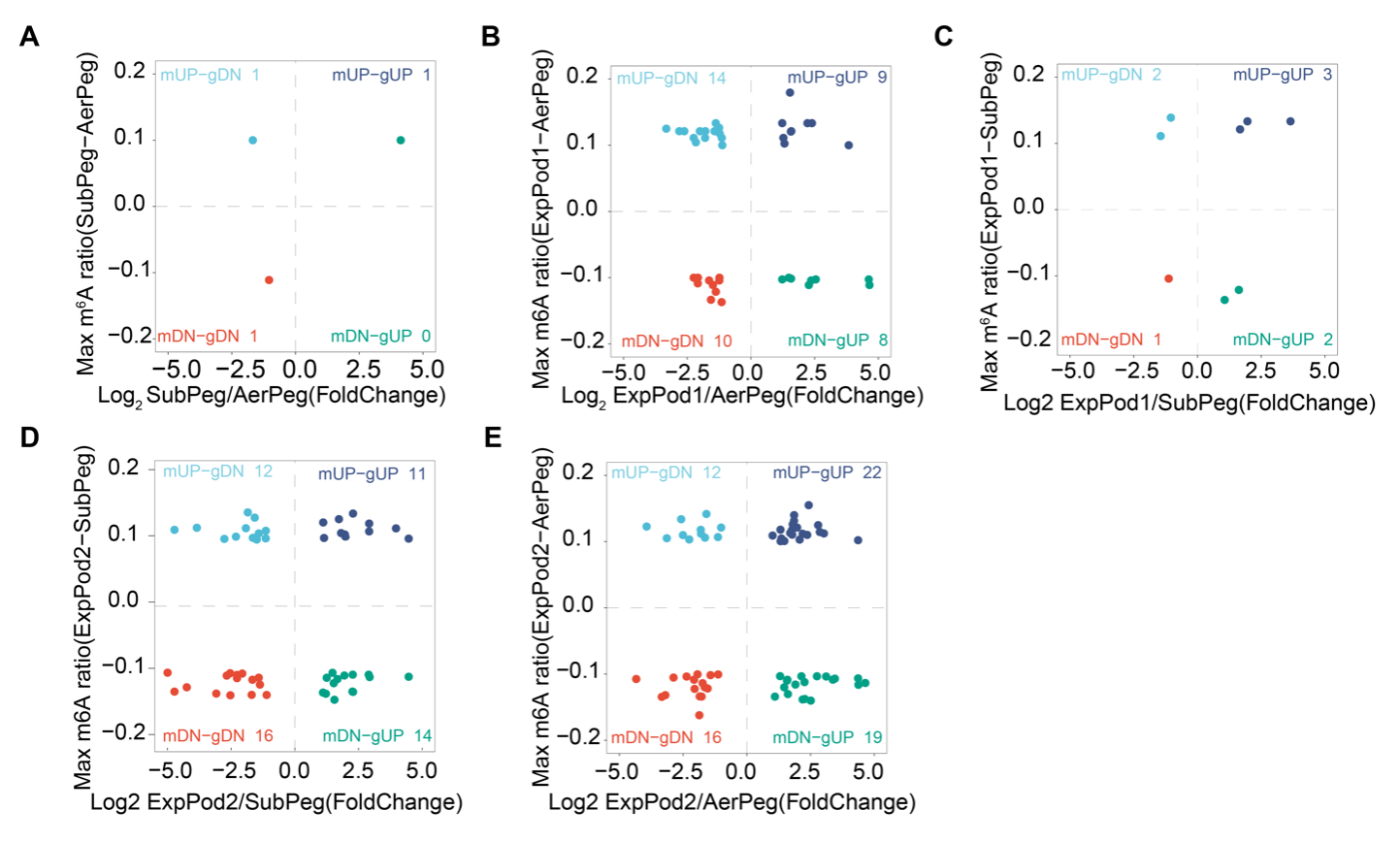


**Supplemental Fig. S8 Scatter plots demonstrate pairwise comparisons of m^6^A ratios versus gene expression. A** SubPeg versus AerPeg. **B** ExpPod1 versus AerPeg. **C** ExpPod1 versus SubPeg. **D** ExpPod2 versus SubPeg. **E** ExpPod2 versus AerPeg.
